# Within the Enemy’s Camp: contribution of the granuloma to the dissemination, persistence and transmission of *Mycobacterium tuberculosis*

**DOI:** 10.3389/fimmu.2013.00030

**Published:** 2013-02-14

**Authors:** Christopher R. Shaler, Carly N. Horvath, Mangalakumari Jeyanathan, Zhou Xing

**Affiliations:** McMaster Immunology Research Centre, Department of Pathology and Molecular Medicine, McMaster UniversityHamilton, ON, Canada

**Keywords:** tuberculosis, immunopathology, immune regulation, granuloma, bacterial persistence, mycobacteria

## Abstract

Pulmonary tuberculosis, caused by *Mycobacterium tuberculosis (M.tb)* represents a leading global health concern, with 8.7 million newly emerging cases, and 1.4 million reported deaths annually. Despite an estimated one third of the world’s population being infected, relatively few infected individuals ever develop active clinical disease. The ability of the host to remain latently infected while preventing disease is thought to be due to the generation of a robust type 1 immune response in the lung, capable of controlling, but not clearing, *M.tb*. A key feature of the type 1 immune response to *M.tb* is the formation of immune cellular aggregates termed granuloma. The granuloma structure has long been considered a hallmark of host’s protective response toward *M.tb*. Historically, a correlative relationship between granuloma formation/maintenance and bacterial control has been seen in models where disrupted granuloma formation or structure was found to be fatal. Despite this established relationship much about the granuloma’s role in *M.tb* immunity remains unknown. Recent publications suggest that the granuloma actually aids the persistence of *M.tb* and that the development of a necrotic granuloma is essential to person-to-person transmission. Our group and others have recently demonstrated that enclosed within the granuloma is a population of immunologically altered antigen-presenting cells and T lymphocyte populations. Of note, the ability of these populations to produce type 1 cytokines such as interferon-gamma, and bactericidal products including nitric oxide, are significantly reduced, while remaining competent to produce high levels immunosuppressive interleukin-10. These observations indicate that although the chronic granuloma represents a highly unique environment, it is more similar to that of a tumor than an active site of bacterial control. In this review we will explore what is known about this unique environment and its contribution to the persistence of *M.tb.*

## INTRODUCTION

Pulmonary tuberculosis (TB) caused by infection with *Mycobacterium tuberculosis (M.tb) *is the leading cause of death due to a bacterial pathogen and is responsible for 1.4 million deaths annually, latently infecting one third of the world’s population (WHO, 2012). Despite the magnitude of individuals infected, the rate of mortality is relatively low with approximately 90% infected individuals controlling, but not clearing, *M.tb *(WHO, 2012). The ability of the host to “control” *M.tb *infection encompasses a number of immunological processes designed to restrain bacterial dissemination and persistence, and reduce person-to-person transmission. The classical hallmark of anti-TB host defense is the formation of type 1 immune granuloma in the lung. Historically, the granuloma has been perceived as essential to anti-TB host defense as the host is incapable of sterile clearance and thus is forced to segregate the infected cells as a means to preserve itself. However, experimentally little is known of the role of the granuloma in bacterial control.

### THE HISTORICAL VIEW OF MYCOBACTERIAL GRANULOMA

First described in 1679, pathologists discovered unique structures in the lungs of TB patients (de le Boe, 1679; reviewed in Ramakrishnan, 2012). These structures were then termed tubercles and represent what we now know as granulomas. Commonly, it was observed that persons who had died of TB had a large number of these distinct pathological lesions, and the presence of tubercles became an associated hallmark of active TB disease. It was not until 1884 that tubercles were also characterized in individuals who had died from diseases other than TB. Upon post-mortem examination, a number of these individuals had lung lesions (granulomas) containing live TB bacilli, giving the first indication that TB latency may relate to the formation of granuloma (Dejerine, 1884). However, upon further microbiological examination, it was revealed that live bacilli persisted not only within the granuloma itself, but also in the surrounding lung tissue, albeit to a lesser degree (Wang, 1916; Opie and Aronson, 1927; Robertson, 1933; Feldman and Baggenstoss, 1938, 1939). It was around this time that the protective view of the granuloma began to gain public acceptance and it was proposed that the recruitment of activated lymphocytes and the formation of a lymphocytic cuff served to wall-off infected macrophages as a means of limiting dissemination. However, the role of granuloma in TB has remained enigmatic largely because of the unavailability of reliable animal models and appropriate techniques to observe the dynamic process of granuloma evolution. Although different experimental models (mice, guinea pig, rabbit, cattle, and macaque) have been developed, only cattle and macaque monkeys form the type of granuloma that closely resembles those seen in humans (Capuano et al., 2003; Flynn et al., 2003; Tsai et al., 2006; Hunter et al., 2007; Via et al., 2008). Despite being extensively used as a model of TB, the murine granuloma lacks many of the unique characteristic features of the human granuloma including centralized necrosis, giant multinuclear cells, and a defined “lymphocytic cuff” (Rhoades et al., 1997). Contrasting the classical notion of its protective role, a number of recent studies have demonstrated unaltered bacterial control even in the absence of granuloma formation, strongly arguing against the granuloma being essential to bacterial restriction (Johnson et al., 1998; Scott and Flynn, 2002; Pearl et al., 2004). Moreover, it is now known that the *Mycobacterium* can significantly alter the immune environment of the granuloma as means to facilitate its persistence (Ly et al., 2007; Scott-Browne et al., 2007; Marino et al., 2010; Castano et al., 2011; O’leary et al., 2011). Regardless, the common perception remains that the granuloma serves to limit bacterial growth and prevent dissemination by segregating infected cells, and the role of the granuloma in *M.tb *infection remains an issue of continued debate. In this review we will challenge the traditional view of the function of granuloma, exploring what is known about its progression and maturation, and how this unique environment may in fact contribute to the persistence and transmission of *M.tb.*

## FORMATION OF THE TYPE 1 IMMUNE GRANULOMA DURING *M.tb* INFECTION

**FIGURE 1:**
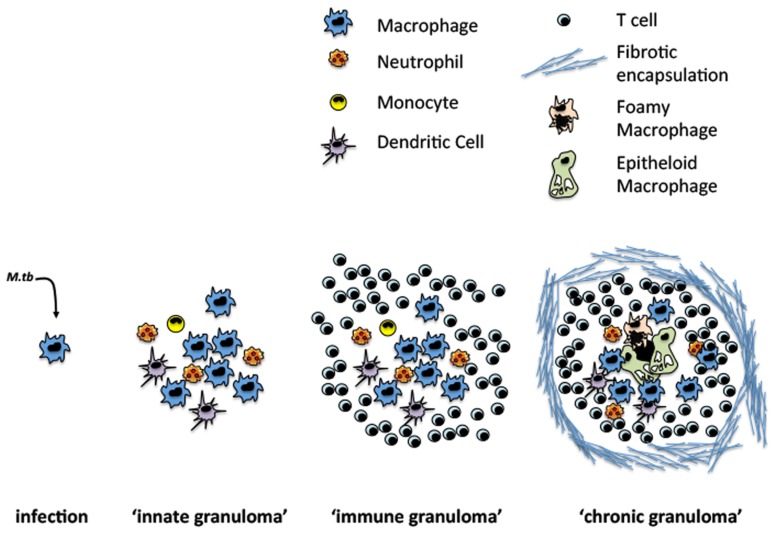
**Evolution of TB granuloma formation**. Following infection sentinel macrophages cells facilitate *M.tb* uptake and the recruitment of other innate and adaptive immune cells.

The formation of granuloma is a dynamic process that begins shortly after infection and continuously evolves over time. Temporally, the granuloma can be divided into three distinct phases: (1) the “innate granuloma,” a loose aggregate composed primarily of recruited macrophages and neutrophils; (2) the “immune granuloma” formed following the emergence of antigen-specific T cells; and (3) the “chronic granuloma,” resulting from distinct morphological changes in granuloma structure (**Figure 1**).

### FORMATION OF THE “INNATE GRANULOMA”

Shortly after aerosol exposure, *M.tb* infects the resident alveolar macrophage (AM) initiating the early inflammatory response. While amplifying the host immune response, the recruitment of innate immune cells inadvertently provides a large number of new targets for *M.tb* to infect and is thought to contribute to the early dissemination of *M.tb *(Doenhoff, 1997; Davis and Ramakrishnan, 2009). Augmenting this problem, the infected AM is unable to kill internalized mycobacteria due to impaired phagolysosome fusion, a process essential to the destruction of the phagocytosed bacteria (Welin et al., 2011). The efficiency by which a *Mycobacterium* species arrests phagolysosome fusion is directly attributable to its relative virulence, with highly virulent strains such as *M.tb* almost completely inhibiting fusion (Ferrer et al., 2010). Incapable of killing internalized *M.tb*, infected macrophages secrete an array of pro-inflammatory and chemoattractant cytokines including tumor necrosis factor (TNF), interleukin (IL)-6, and IL-8 which facilitate the recruitment of new macrophages and granulocytes to the site of infection and lead to the formation of the “innate granuloma” (Birkness et al., 2007). This initial recruitment is essential to establishing the macrophage-dominated center of the “immune” granuloma.

### CONTRIBUTION OF *M.tb* TO THE FORMATION OF THE “INNATE GRANULOMA”

Historically the formation of granuloma has been considered to be host-mediated event. Using an *M. marinum* model, real-time microscopic visualization has challenged this notion, revealing that virulent *Mycobacterium* drives the nascent formation of the early granuloma. A number of elegant studies conducted by Ramakrishnan’s group have demonstrated the unique interplay between the *Mycobacterium* and the host immune system in the early stage of granuloma formation. To this end, the early release of 6 kDa early secretory antigenic target (ESAT-6) led to the activation of the epithelium, which facilitated the recruitment of macrophages to the site of infection through inducing the production of matrix metalloproteinase-9 (MMP-9) (Davis and Ramakrishnan, 2009; Volkman et al., 2010). In murine models, *M.tb* was also found to drive MMP-9 expression. Thus either broad MMP inhibition or MMP-9-specific depletion delayed granuloma formation, resulting in impaired macrophage recruitment to the site of infection and reduced granuloma size (Taylor et al., 2006). These findings indicate that *M.tb* may actually promote granuloma formation and utilizes the structure for its own benefit. Furthermore, it was demonstrated that virulent mycobacteria can utilize the innate granuloma as means of recruiting target cells allowing for the early dissemination of mycobacteria throughout the host (Davis and Ramakrishnan, 2009). This view is supported further by the observation that the early granuloma is not a static environment and there is a significant movement of antigen-presenting cell (APC) populations into and out of the early granuloma (Chiu et al., 2004; Egen et al., 2008, 2011; Schreiber et al., 2011).

### FORMATION OF THE “IMMUNE GRANULOMA”

Following innate activation, dendritic cells are recruited to the lung and transport mycobacteria or mycobacterial antigens to the mediastinal lymph node (MLN). Within the MLN antigen-loaded APCs activate antigen-specific T cells. Due to the nature of *M.tb* infection, the majority of bacilli and antigen reside within an endosome, and are most efficiently loaded onto major histocompatibility complex (MHC) class II (Mogues et al., 2001; Anis et al., 2008; Yahagi et al., 2010). The loading of MHC class II facilitates the priming of Th1 interferon-gamma (IFN-γ)-secreting T cells, which rapidly home to the lung. While the dominant subset of T cells are CD4^+^, cross-presentation also allows for the strong induction of CD8^+^ T cells, collectively generating a type 1 polarized adaptive immune response (Winau et al., 2006). Although outside the scope of this review, it should be noted that there is a substantial lag period between *M.tb* infection and the initiation of antigen-specific T cell responses (reviewed in Shaler et al., 2012).

The continuous production of chemokines by infected lung APCs efficiently recruits newly primed T cells into the lung. Once in the lung, recruited T cells surround and wall-off infected macrophages, activate them for enhanced bactericidal function, and physically limit their mobility to restrain bacterial dissemination. Indeed, the arrival of effector T cells and the establishment of the classical “immune granuloma” is associated with a plateau in bacterial growth (Mogues et al., 2001). While the prevailing immune response generated following *M.tb* infection is highly similar between the mouse and man, the structural formation of the “immune granuloma” differs significantly. In mice, many of the hallmark features of the human granuloma are missing, and thus the knowledge from murine granuloma research should be interpreted with caution. Despite these limitations, animal models have provided significant insight, and have been invaluable in delineating the stages of granuloma formation.

### THE CONTROVERSIAL REQUIREMENT OF THE “IMMUNE GRANULOMA” IN LIMITING BACTERIAL DISSEMINATION

In spite of the traditional protective view of granuloma, it has recently been revealed that the immune environment within granuloma is more conducive to *M.tb* persistence than its elimination. Despite the fact that animal models do not accurately replicate human granuloma structures, several murine studies have provided invaluable insight into the role of granuloma in preventing *M.tb *dissemination control. Early studies demonstrating the role of critical cytokines such as TNF and IFN-γ perpetuated the notion that the granuloma was essential for bacterial segregation and limiting bacterial growth in the lung as the absence of either cytokine led to ill-formed granuloma and increased bacterial infection (Flynn et al., 1998; Kaneko et al., 1999; Algood et al., 2005; Beham et al., 2011; Gallegos et al., 2011). Moreover, any loss of CD4^+^ T cell functionality results in a loss of the granuloma structure and extensive bacterial dissemination is seen in both man and mouse. While essential to the control of *M.tb*, the role of the CD4^+^ T cell in the granuloma’s structure is somewhat species-dependent. Specifically, in humans, T cells surround and wall-off infected macrophages, and do not infiltrate the granuloma, but rather form a defined lymphocytic cuff. Conversely, murine CD4^+^ T cells associate directly with infected cells infiltrating throughout the granuloma forming lymphocytic aggregates or pseudo-granulomas (Mogues et al., 2001; Tsai et al., 2006; Hunter et al., 2007; Via et al., 2008; Gallegos et al., 2011; Geldmacher et al., 2012). Nevertheless, while the loss of CD4 T cell-mediated immunity is detrimental to the host it is impossible to separate the relative contribution of the two processes to the impaired bacterial control: loss of Th1-mediated immunity and loss of granuloma structure, these studies suggest a critical role for the granuloma in preventing bacterial dissemination (Saunders et al., 2002; Segovia-Juarez et al., 2004). The essential role of CD4 T cells to the control of mycobacterial growth has largely been attributed to their potent IFN-γ production and subsequent macrophage activation. While macrophage activation is essential to the control of *M.tb *in mouse and man, the role of nitric oxide has been an issue of some debate. Historically, studies have shown that human macrophages do not produce nitric oxide to the same degree *in vitro *as those isolated from mice (Aston et al., 1998). Recently, however, several groups have demonstrated that human macrophages from TB infected patients, as well as human macrophage cells lines are capable of inducing iNOS and producing nitric oxide in response to *M.tb* antigen (Rich et al., 1997; Jagannath et al., 1998; Dlugovitzky et al., 2000). Indeed, it appears that while the timing of nitric oxide and its role in TB control may differ somewhat between mouse and man, nitric oxide remains important in both species.

Recent studies suggest that the granuloma may be dispensable for preventing bacterial dissemination and may actually contribute to *M.tb* persistence. Moreover, in the absence of intracellular adhesion molecule-1 (ICAM-1), there is also a failure of granuloma formation, and despite this defect, mice are protected for the first 90 days post-infection, with no increase in bacterial growth compared to wildtype mice within this time frame (Johnson et al., 1998). Similarly, zebrafish models have shown that in the absence of early granuloma formation, there is no defect in the ability of the host to limit bacterial replication and dissemination, and that the granuloma may actually facilitate early dissemination (Volkman et al., 2004, 2010). Furthermore, in the absence of IL-27 in mice, there is a substantial defect in the ability of the host to form granuloma in response to *M.tb* infection, and yet infected mice exhibit markedly enhanced bacterial control when compared to their wildtype counterparts (Pearl et al., 2004). Indeed, recent studies indicate that granuloma does not always function to limit bacterial dissemination. For example, C-C chemokine receptor type 2 (CCR2) deficient mice form exaggerated granuloma structures when infected with *M.tb* and paradoxically have a decreased capacity to control bacterial growth (Scott and Flynn, 2002).

While it remains debatable whether the granuloma is required for bacterial control, growing evidence supports the notion that the fate of *M.tb* within the granuloma is situation-dependent. For example, the initial inoculum size may influence the number of macrophages and granulocytes that are recruited to the site of infection. If a large number of cells are initially recruited, spatially it becomes difficult for effector T cells to interact with the infected cells residing at the core of the granuloma limiting their ability to activate these centralized macrophages to kill internalized *M.tb *(Segovia-Juarez et al., 2004). In comparison, a small initial inoculum size infects a small number of cells at the core of granuloma, which may increase the likelihood of interaction of infected cells with effector T cells (Segovia-Juarez et al., 2004). Based on this notion, it is tempting to speculate that a stronger initial innate immune response may perpetuate the infection and limit the host’s ability to eliminate *M.tb*. Therefore, a small-size granuloma may favor host defense whereas a relatively large-size counterpart may favor the persistence of mycobacterial bacilli, regardless of the magnitude of T cells generated. The current limitation to diagnostic imaging makes studying the evolution of the granuloma in latently infected humans difficult and as a consequence, little is known about how its structure changes over the course of infection in otherwise healthy individuals.

### THE “IMMUNE GRANULOMA”: A NICHE FOR BACTERIAL PERSISTENCE

Regardless of whether the granuloma functions to limit bacterial dissemination, much evidence suggests that *M.tb* is especially adept at altering the immune response within the granuloma, creating a uniquely suppressed environment largely through the induction of IL-10 (de Waal Malefyt et al., 1993; Chiu et al., 2007, 2008; Higgins et al., 2009; Marino et al., 2010; Redford et al., 2010; O’leary et al., 2011; Shaler et al., 2011). Functionally, the infected macrophages within the granuloma are altered, showing a reduced capacity to produce bactericidal products such as nitric oxide, while showing enhanced IL-10 production (de Waal Malefyt et al., 1993; Chiu et al., 2007, 2008; Higgins et al., 2009; Marino et al., 2010; Redford et al., 2010; O’leary et al., 2011; Shaler et al., 2011). Interestingly, while the macrophage populations of the granuloma have reduced bactericidal function, they continue to produce large amounts of chemokines facilitating the continuous recruitment APC populations into the granuloma (Schreiber et al., 2010; Shaler et al., 2011). Recent studies utilizing *intravital* microscopy have revealed significant movement of inflammatory APCs both into and out of the granuloma (Schreiber et al., 2011). It is this movement of infected APCs that has been speculated to facilitate the early dissemination of *M.tb*. Likewise, human granuloma contains a high frequency of foxp3+ T regulatory cells (Rahman, 2009). In addition, murine studies have confirmed that T cells residing within the granuloma display a highly altered, and functionally suppressed phenotype. Despite the central role of IL-10 in suppressing T cell and macrophage activation within the granuloma, IL-10 neutralization or infection of IL-10 knockout (KO) mice results in only marginally reduced bacterial loads (de Waal Malefyt et al., 1993; Jacobs et al., 2002; Chiu et al., 2007; Higgins et al., 2009). Given *M.tb’s* long evolution with humans, it is not surprising that *M.tb* targets multiple pathways to interrupt the host immune response. Moreover, while conventionally immune suppression would appear to benefit only the pathogen, the induction of IL-10 may actually be a host-mediated event required to limit unwanted immunopathology.

### THE “CHRONIC GRANULOMA”: A DYNAMIC INTERPLAY BETWEEN PERSISTING *M.tb* AND THE HOST IMMUNE RESPONSE

Following the establishment of the “immune granuloma,” a period of immune quiescence is established. It is during this stage that chronic immune activation leads to significant alterations in the morphology and functionality of the granuloma, many of which are not typically seen in mice. Post-mortem studies of latently infected humans and non-human primates have revealed that the evolution of the granuloma structure is highly divergent, not only between individuals, but even within a single individual (Capuano et al., 2003; Flynn et al., 2003; Barry et al., 2009). Typically, within an infected individual, a spectrum of granuloma structures are seen. For instance, both the fully calcified lesions containing no bacilli and the fibrotically encapsulated necrotic granulomas containing large numbers of live bacteria can be seen in the lung of the same individual, indicating that the evolution of the granuloma is a highly dynamic process (Capuano et al., 2003; Flynn et al., 2003; Barry et al., 2009).

While the granuloma has long been believed to be a protective host’s response, it is now acknowledged to result from a dynamic and continuous interplay between the host’s immune response and persisting *M.tb.* The continuous “battle” between chronic immune activation and bacterial persistence causes infected macrophages to adopt an irregular epithelial-like or “epithelioid” appearance, and to fuse together forming multinucleated giant cells in the core of granuloma (Hunter et al., 2007; Hunter, 2011). Indeed, the virulent *Mycobacterium* has been shown to induce macrophage cell death frequently throughout the course of infection, which several studies have shown to be a potential mechanism of gaining access to new host macrophages (Davis and Ramakrishnan, 2009). Newly recruited macrophages quickly phagocytose the dead bodies and become saturated with mycobacteria and lipids (Peyron et al., 2008). These lipid-rich macrophages that accumulate within the granuloma are known as foamy macrophages due to their distinct appearance, and are now recognized as a major contributing factor in the persistence of *M.tb* (Peyron et al., 2008). Recent studies have shown that foamy macrophages isolated from humans have lost key functions including their ability to phagocytose and to produce essential bactericidal agents such as nitric oxide (Peyron et al., 2008). Moreover, the polarization of macrophage populations within the granuloma are thought to shift from being a classically activated (M1) population toward that of an alternatively activated (M2), with reduced bactericidal capacity (Redente et al., 2010). Thus, such foamy macrophages have been proposed to function as the reservoirs of *M.tb* whereby the bacterium is able to successfully manipulate the infected macrophage into not only a safe haven but also a source of nutrients required for the synthesis of its cell wall and replication.An infected host typically houses a highly heterogeneous mixture of granuloma types ranging from large necrotic granulomas containing large amounts of bacilli, to completely calcified structures devoid of any detectible bacteria (Turner et al., 2003; Hunter et al., 2007; Hunter, 2011). In the later stages of granuloma evolution, fibrotic encapsulation can be seen in cases of both active and latent infection. Currently, it is unclear whether encapsulation functions to prevent bacterial escape, or to limit immune infiltration into the granuloma. Although complete mycobacterial clearance is rarely seen, latently infected humans display the evidence of healed granulomas, marked by central calcification in conjunction with fibrotic encapsulation containing no detectible bacilli (Opie, 1917). Despite the observed absence of bacteria within highly calcified granulomas, it is currently unclear whether this represents immune-mediated clearance, or simply a structural artifact left behind following *M.tb* escape.

### *Mycobacterium tuberculosis* FACILITATES PERSON-TO-PERSON TRANSMISSION THROUGH ALTERATIONS TO THE GRANULOMA

**FIGURE 2:**
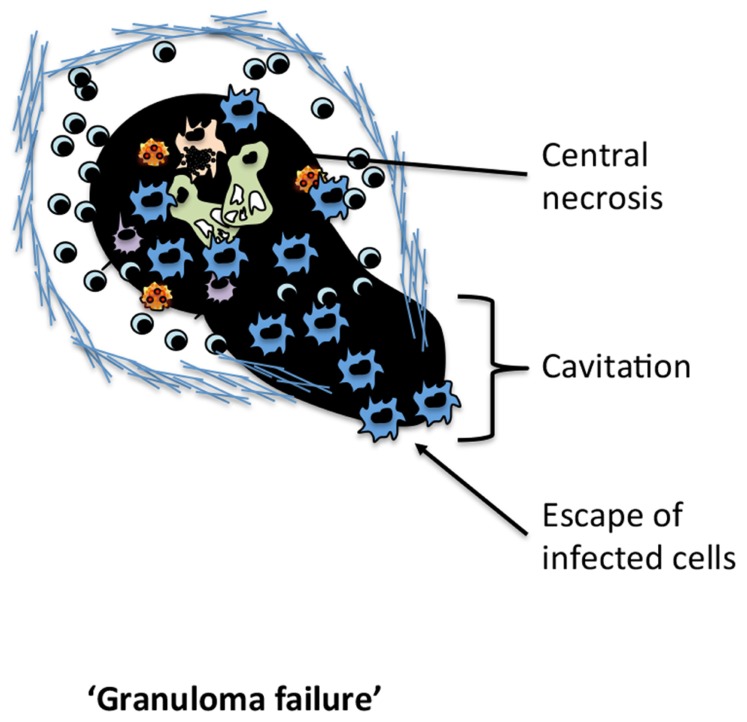
**The failure of the TB granuloma**. The characteristic changes associated with the disintegration of the TB granuloma required for dissemination.

Despite the attempts of the host to contain *M.tb* within the granuloma, as the infection progresses, the majority of individuals will develop granulomas with a necrotic focus formed due to the caesation of the macrophage infused center (Kim et al., 2010; **Figure 2**). This eventual necrosis of the granuloma is now accepted as a necessary event in facilitating the transmission of *M.tb *by disrupting the lung structure and allowing *M.tb* to gain access to the major airways. It should be noted that, in addition to person-to-person transmission, the active granuloma leaking bacteria into the airways may also allow for intrapulmonary dissemination (Cardona, 2009, 2010; Cardona and Ivanyi, 2011). It is therefore likely that the heterogeneity in granuloma structure seen in different lung regions of the same host represents different evolutionary timelines. Although much remains to be understood, it is clear that the evolution of granuloma is the result of the dynamic interplay between persisting mycobacteria and the host immune response, continuously evolving throughout the course of *M.tb* infection. Interestingly, *M.tb* may actually utilize the host immune response to facilitate the structural changes required to facilitate person-to-person transmission. In line with this, while essential to preventing bacterial dissemination, paradoxically IFN-γ-producing Th1 cells may also play an integrate role in facilitating bacterial transmission (Ehlers et al., 2001). The ability of *M.tb* to manipulate the host immune response as means to facilitate central granuloma necrosis and facilitate its transmission while deterring immune-mediated bacterial clearance is a remarkable but poorly understood feature of *M.tb*. It is important to note that the processes of bacterial dissemination within a host, and transmission between hosts may be independently regulated. For instance, clinically it has been observed that despite exaggerated bacterial burdens and extensive dissemination, HIV-AIDS individuals co-infected with *M.tb *transmit *M.tb *person-to-person far less efficiently (Doenhoff, 1997; Ledru et al., 1999; Corbett et al., 2003; Glynn et al., 2008). The inability of the HIV-infected host to spread *M.tb* has been attributed to a failure of *M.tb* to drive central granuloma necrosis and cavitation, and the transport of bacilli to the airway. These observations argue that *M.tb* utilizes the necrotic granuloma as a portal for person-to-person transmission.

While it is well-known that changes to the granuloma structure are required for bacterial transmission, it is currently unknown whether the host or bacteria are responsible for these changes. Recently, studies have examined the granuloma at early and chronic stages of disease revealing a dramatic shift in the genes expressed by both *M.tb* and the host immune response. Notably, within the granuloma, the host immune response shifts from predominately pro-inflammatory during the early phases of infection, to immunosuppressive during the chronic stages (Karakousis et al., 2004; Ly et al., 2007; Mehra et al., 2010). Coincidentally, *M.tb* expresses a defined set of genes that function to facilitate immune activation, while simultaneously expressing enzymes to combat immune-mediated clearance (Rohde et al., 2012). This is later followed by a shift in gene expression thought to facilitate immune senescence within the granuloma, allowing for *M.tb’s* persistence (Rohde et al., 2012). While traditionally *M.tb *is thought to lie dominant, it has recently been demonstrated that throughout the course infection *M.tb* will periodically “awaken,” up-regulating a number genes and sample the immune environment (Karakousis et al., 2004). This sampling allows *M.tb *to identify the optimal conditions for facilitating person-to-person transmission. Moreover, during the caseous stage of granuloma formation there is a further shift in the genes expressed by *M.tb* with a significant up-regulation of genes associated with lipid metabolism (Kim et al., 2010). Notably, most mycobacterial species are rich in immunomodulatory lipids, which play a central role in immune evasion. Intriguingly, the distribution and release of certain lipids by *M.tb* varies significantly over the course of infection, providing a means by which *M.tb *directs the host immune response. To this end, *M.tb* can release toxic lipids and generate targeted tissue damage. The generation of central necrosis is essential to facilitating cavitation and promoting *M.tb *transmission. Trehalose 6,6′-dimycolate (cord factor) has potent cytotoxic effects and has been implicated in the generation of central necrosis of the granuloma and the transmission of *M.tb* (Hunter et al., 2006; Kim et al., 2010). Recently, it has been shown that neutrophils and AMs recognize mycobacterial cord factor through their surface c-type lectin receptor, mincle (Behler et al., 2012; Lee et al., 2012). The engagement of mincle leads to a pro-inflammatory cytokine pathway that aids in the early cellular recruitment and control of mycobacteria (Behler et al., 2012; Lee et al., 2012). Interestingly, however, the proportion of cord factor varies greatly throughout the course of infection with its synthesis heavily up-regulated by *M.tb *during the development of central necrosis and cavitation (Hunter et al., 2006; Kim et al., 2010). Indeed, studies have linked the amount of cord factor released by *M.tb* to the extent of necrosis and cavity formation (Hunter et al., 2006; Kim et al., 2010). Moreover, previous studies have documented mincle as a key receptor in the detection of necrosis and the development of an inflammatory response upon tissue damage (Yamasaki et al., 2008). Given that cord factor is known for its cytotoxic effects, one may speculated that engaging mincle may be central to the development of a pro-inflammatory response capable of aiding the formation of cavitation within the granuloma. Utilizing the host immune machinery *M.tb* facilitates the necessary structural changes to ensure its own transmission, which occurs at a time when the immune system is most vulnerable.

## CONCLUDING REMARKS

While the true nature of the granuloma still remains to be defined, it is now clearly evident that the granuloma is not just a host-mediated entity of segregation and rather, it is a dynamic battlefield bearing the scars left both by the pathogen and the host immune response. While it may have been originally destined to restrain bacterial dissemination, *M.tb* efficiently hijacks the granuloma to provoke the generation of an immunologically sheltered niche to reside within and persist until the situation is favorable to bacterial transmission.

## Conflict of Interest Statement

The authors declare that the research was conducted in the absence of any commercial or financial relationships that could be construed as a potential conflict of interest.
